# Prevalence of Selected Chronic, Noncommunicable Disease Risk Factors in Jordan: Results of the 2007 Jordan Behavioral Risk Factor Surveillance Survey

**Published:** 2011-12-15

**Authors:** Mohannad Al-Nsour, Meyasser Zindah, Adel Belbeisi, Raja Hadaddin, David W. Brown, Henry Walke

**Affiliations:** Ministry of Health; Ministry of Health, Amman, Jordan; Ministry of Health, Amman, Jordan; Ministry of Health, Amman, Jordan; The Brown Consulting Group, Charlotte, North Carolina; Centers for Disease Control and Prevention, Atlanta, Georgia

## Abstract

**Introduction:**

Noncommunicable diseases (NCDs) are the leading cause of illness and death in Jordan. Since 2002, the Jordan Ministry of Health, in cooperation with the World Health Organization and the Centers for Disease Control and Prevention, established the Jordan Behavioral Risk Factor Surveillance Survey to collect information on many of the behaviors and conditions related to NCDs. The objectives of this study were to describe the prevalence of selected NCD risk factors and the relationship between body mass index and selected health conditions among a nationally representative sample of Jordanian adults aged 18 years or older.

**Methods:**

We used a multistage sampling design to select 3,688 households, from which we randomly selected and interviewed 1 adult aged 18 years or older. A total of 3,654 adults completed the survey. We randomly selected a subsample of 889 interviewed adults and invited them to visit local health clinics for a medical evaluation; we obtained measurements, including fasting blood glucose and blood lipids, from 765 adults. Data were collected between June 1, 2007, and August 23, 2007.

**Results:**

Nearly one-third of participants smoked cigarettes, 18% reported having been diagnosed with high blood pressure, and 10% reported frequent mental distress. Compared with survey participants who did not participate in the medical evaluation, those who participated were more likely to self-report high blood pressure, high blood cholesterol, and diabetes and report lower levels of health-related quality of life. Among participants of the medical evaluation, an estimated 11% reported they had been diagnosed with diabetes by a health professional, and 19% were diagnosed with diabetes according to laboratory testing. Approximately one-third of participants of the medical evaluation were either overweight (30%) or obese (36%). In the fully adjusted model, obese participants of the medical evaluation were nearly 3 times as likely to have high blood pressure and more than 2 times as likely to have high blood cholesterol as normal-weight participants.

**Conclusion:**

Diabetes, high blood pressure, high cholesterol, and obesity are a public health concern in Jordan. Adequate and continuous monitoring of NCD risk factors in Jordan is needed, and the surveillance findings should be used in health promotion and disease prevention activities.

## Introduction

Jordan, as in many middle-income countries, has witnessed a demographic change. In 2007, the infant mortality rate decreased to 19 per 1,000 live births, and life expectancy increased for men (71.6 y) and women (74.4 y) (1). Jordan is also undergoing an epidemiologic transition; the burden of infectious diseases has lessened, but the burden of noncommunicable diseases (NCDs) has increased. NCDs are the leading cause of death in Jordan; more than one-third of deaths are attributed to cardiovascular diseases and 14% to cancer (2,3). According to World Health Organization projections, NCDs will be responsible for two-thirds of deaths in the eastern Mediterranean region by 2030 (4). NCD risk factors such as smoking, physical inactivity, obesity, and unhealthful diets are now serious public health problems in Jordan (5,6).

Beginning in 2002, the Jordan Ministry of Health (MOH), in cooperation with the Jordan Department of Statistics (the organization responsible for conducting national surveys in Jordan), the World Health Organization, and the Centers for Disease Control and Prevention, established the Jordan Behavioral Risk Factor Surveillance Survey (BRFSS) to collect data on health risk behaviors, clinical preventive health practices, and health care access that are associated with leading causes of illness and death in Jordan. The Jordan BRFSS also collects data on many of the behaviors and conditions related to NCDs and is useful for monitoring and evaluating the effectiveness of public health programs. The Jordan BRFSS, a in-person household interview survey that uses a national sampling frame, was conducted a second time in 2004. Results of the 2002 and 2004 Jordan BRFSS are published elsewhere (7,8).

The objectives of this study were to describe the prevalence of selected NCD risk factors and the relationship between body mass index (BMI) and selected health conditions among a nationally representative sample of Jordanian adults aged 18 years or older.

## Methods

The Jordan MOH conducted its third BRFSS in 2007. We used a multistage sampling design to select households in which the survey was administered. We used the 2004 Jordan census to identify census enumeration blocks for the master sampling frame, and we selected households from a sample of blocks, or primary sampling areas. This sampling frame was stratified by governorate, major city, other urban area, and rural area into 30 strata that fit within 3 regions, north, middle, and south. Geographic ordering of the blocks in the frame provided implicit stratification. In each stratum, we systematically selected a sample of 461 blocks with probability proportional to Jordan's total population (Appendix). We selected 8 households from each block. In each household, we randomly selected 1 adult aged 18 years or older and interviewed that person in Arabic. We conducted interviews between June 1, 2007, and August 23, 2007.

Of 3,688 households selected, 3,654 adults (99%) were successfully interviewed. The survey instrument (available on request) included questions on demographics (eg, sex, age, educational status), health status, health care access, tobacco use, physical activity, nutrition, hypertension and cholesterol awareness, and prevalence of heart disease, diabetes, and asthma.

We defined respondents who smoked 100 cigarettes in their lives and who currently smoked as current smokers. We assessed participation in moderate physical activity with the question, "Do you do any moderate-intensity sports, fitness or recreational (leisure) activities that cause a small increase in breathing or heart rate such as brisk walking and lifting light and moderate weights for at least 10 minutes continuously?" To assess consumption of fruits and vegetables we asked, "How many cups of fresh or cooked vegetables did you have yesterday?" and "How many cups of fruits or fresh juices did you have yesterday?" We considered people who responded yes to the question, "Have you ever been told by a health professional that you have high blood pressure?" to have hypertension. We considered people who responded yes to the question, "Have you ever been told by a health professional that your blood cholesterol is high?" to have high blood cholesterol. We considered people who responded yes to the question, "Have you ever been told by a health professional that you have diabetes?" to have diagnosed diabetes. Type of diabetes was not assessed. Women who reported having gestational diabetes only were considered not to have diabetes.

We asked respondents the following questions related to health-related quality of life (HRQOL): "Would you say in general your health is excellent, very good, good, fair, or poor?" On the basis of the response to this question, we defined a dichotomous variable for fair or poor self-rated health status. We also asked respondents, "Now, thinking about your physical health, which includes physical illness and injury, for how many days during the past 30 days was your physical health not good?" and "Now, thinking about your mental health, which includes stress, depression, and problems with emotions, for how many days during the past 30 days was your mental health not good?" We did not ask respondents for specific underlying reasons of any reported unhealthy days. These questions and their construct validity are described elsewhere (9,10). We calculated overall unhealthy days as the sum of physically and mentally unhealthy days, not to exceed 30 days. We defined a dichotomous HRQOL variable as fewer than 14 or 14 or more unhealthy physical days, unhealthy mental days, and unhealthy days (mental or physical). A total of 14 unhealthy days is a meaningful cut point for participants reporting substantially impaired HRQOL.

All questions were translated from English into Arabic and then back translated to ensure accuracy. To ensure consistency, we conducted pilot tests of the Jordan BRFSS for 2002, 2004, and 2007 under realistic field conditions and used the same trained interviewers who were recruited to conduct the actual survey to implement the pilot tests. The testing process accounted for all survey activities: approaching potential participants, seeking and obtaining informed consent, making arrangements/appointments for data collection, preparing the site, collecting all data, identifying follow-up cases, and avoiding double data entry. Twenty male and female participants of different educational and socioeconomic levels and varied ages were used for each pilot test. We summarized participants' comments into a single report and made modifications to the survey instrument, ensuring intended meanings were retained.

To compare self-reported health information to actual medical measurements, we selected a sample of 116 of the total 461 blocks and invited survey respondents to participate in a standard medical examination. Participants completed a consent form, and the study design was approved by the Jordan MOH. Of the 889 survey respondents who were invited to participate in the medical examination, 765 (86%) agreed to participate. Participants were evaluated at local health clinics, where height, weight, waist circumference, and blood pressure measurements were obtained. A fasting blood sample was obtained from each participant and sent to a central laboratory where total cholesterol and blood glucose were measured. Standardized training was provided to the attending physicians of the participating local health clinics, and all participating physicians used the same standard equipment for blood testing and for measuring height and weight.

For participants of the medical examination, we computed BMI as weight divided by the square of height (kg/m^2^). Participants were classified as normal weight (BMI <25.0), overweight (BMI 25.0-29.9), and obese (BMI ≥30.0). We defined high blood pressure as 140/90 mm Hg (systolic/diastolic), high blood cholesterol as ≥240 mg/dL, impaired fasting glucose as 100 mg/dL to 125 mg/dL, and diabetes as ≥126 mg/dL. We considered the presence of antihypertensive medications for high blood pressure, lipid lowering medications for high blood cholesterol, and insulin or oral hypoglycemic medication for impaired fasting glucose and diabetes for these classifications.

For respondents who participated in the medical evaluation, we estimated the relative odds of overweight and of obesity associated with selected health risk factors by using logistic regression analysis adjusted for sex, age, education, smoking, physical activity, and fruit and vegetable consumption. We used STATA statistical software (STATA Corporation, College Station, Texas) in all analyses to accommodate the complex survey sampling design.

## Results

Overall, nearly one-third of participants smoked cigarettes, 38% engaged in moderate physical activity, and 17% consumed 5 or more servings of fruits and vegetables per day (Table 1). Approximately 18% of participants reported having been diagnosed with high blood pressure, 7.5% with high blood cholesterol, and 10% with diabetes, and 10% reported frequent mental distress. Approximately 78.8% (standard error [SE], 0.80%) of respondents had ever been tested for high blood pressure, and 37.5% (SE, 0.88%) had ever had their cholesterol levels checked (data not shown). Participants who agreed to participate in the medical examination were more likely to be female, older, and with lower educational levels than those who participated in the household interview only. They also were more likely to have been diagnosed with high blood pressure, high blood cholesterol, or diabetes and have lower levels of health-related quality of life. Prevalence of overweight was 30.5% and prevalence of obesity was 36.0%, based on measured weights and heights of participants of the medical evaluation (Table 1).

The percentages of participants in the medical evaluation who had high blood pressure, high blood cholesterol, and undiagnosed diabetes were high (Table 2). Approximately 11% of participants reported they had been diagnosed with diabetes, compared with 16% who were diagnosed by laboratory testing and 19% who were diagnosed by laboratory testing or current use of insulin or an oral hypoglycemic medication. Approximately 23.9% (SE, 1.75%) of participants had impaired fasting glucose (data not shown).

After adjusting for the full model, compared with adults of normal weight, obese adults were nearly 3 times as likely to have high blood pressure, more than 2 times as likely to have high blood cholesterol, and 1.7 times as likely to report fair or poor health (Table 3).

## Discussion

Our study shows a high prevalence of overweight and obesity, hypertension and diabetes among Jordanian men and women. Consistent with previous findings from Jordan (6), our data show that a high percentage of people with diabetes are not diagnosed. The high prevalence of diabetes and obesity coupled with high levels of undiagnosed conditions and smoking, particularly among men (11), indicate the need for immediate implementation of programs to prevent and control NCDs in Jordan.

Higher-than-normal BMI and weight gain are risk factors for diabetes (12), and other studies have indicated that changes in BMI at the population level foreshadow changes in diabetes prevalence (13,14). Obesity and diabetes usually are preventable. Previous studies have demonstrated that changes in lifestyle can prevent diabetes and obesity in selected groups of adults who are at high risk (15,16).

The diagnosed prevalence of diabetes among Jordanian adults aged 18 years or older increased from 6.4% in 2002 to 7.5% in 2004 and to 9.4% in 2007 (7,8). Between 2002 and 2004, the self-reported prevalence of obesity increased 50%, from 12.8% to 19.5%. The increasing prevalence of obesity and diabetes in Jordan most likely will continue to rise in the years ahead, driven by both population aging (17) and rapid social and environmental changes, unless effective interventions are implemented.

Our finding that Jordanian adults' self-reported prevalence of 14 or more unhealthy mental days has increased, from 5.7% in 2004 (18) to 10.3% in 2007, is interesting. Historically, Jordanians have strong family bonds and a wide social support network. Mental health services, such as counseling, are not widely available or easily accepted. With globalization and the influence of Western culture, the extended family's role is diminishing. The high rates of mental distress in our study may indicate a shift in health needs in Jordan and the area.

Our findings are subject to several limitations. First, the survey is cross-sectional and was not conducted throughout the year; therefore, some of the behaviors that vary seasonally (eg, dietary intake) may not be representative, and cause and effect cannot be determined for the associations between BMI and selected health conditions. Second, some variables were self-reported, which may have resulted in self-report bias. We did not compute measures of agreement between self-reported conditions and those obtained from actual measurements. In contrast to prior estimates of obesity in Jordan, which were based on self-reported weight and height and were, therefore, conservative (19), estimates from the 2007 Jordan BFRSS were based on actual measurements among participant of the medical examination. Although we selected the subsample of survey respondents randomly for medical evaluation from the pool of people participating in the household interview, self-selection bias may have been a factor in the differences noted between the group undergoing medical examination and the larger group that participated in the household interview from which the subsample was selected. Finally, although our survey questions related to behavioral risk factors have documented validity in English, the questions were translated into Arabic; validity of such questions in the Arabic-speaking world have not been studied (20). Studies to develop better measures for mental health in the region are also needed.

Despite these limitations and as NCD^ ^risk factors continue to rise in Jordan, the need remains^ ^for reliable, transparent information, such as that provided by the Jordan BRFSS, to support^ ^evidence-based health policy and programs. The high response rate (95%) to this survey reflects the hospitality of Jordanian culture and the skill of trained interviewers, and the continued implementation of standardized methods in collecting risk factor surveillance data in Jordan facilitates comparisons over time.

The prevalence of NCDs and NCD risk factors in Jordan is high. Adequate and continuous monitoring of NCD risk factors in Jordan is needed, and the surveillance findings should be used in health promotion and disease prevention activities. Programs to monitor and control risk factors and clinical services and a robust health care system are needed to successfully improve NCD outcomes and reduce the burden of disease in Jordan. Reducing the prevalence of NCDs requires a renewed commitment by governmental and nongovernmental institutions, by public health professionals and clinical practitioners, and by communities and individuals to acknowledge the burden of NCDs and the need for timely action. Moreover, stimulating, strengthening, and sustaining regional efforts and programs are necessary to reduce the prevalence of NCDs through coordinated and integrated programs of health promotion and disease prevention. These programs should involve networks for risk factor surveillance, information sharing, capacity building, advocacy, policy development, and collaboration in generating, disseminating, and applying knowledge.

In collaboration with its partners, the Jordan MOH and the Jordan Applied Epidemiology Training Program are developing and implementing a national chronic disease prevention and control plan. This plan will target primary risk factors and behaviors associated with chronic diseases (eg, smoking, overweight, unhealthy diet, physical inactivity) and call for collaboration among all governmental ministries, nongovernmental organizations, and the private sector.

## Figures and Tables

**Table1.** Participant Characteristics, Household Interview and Medical Examination, Behavioral Risk Factor Surveillance Survey, Jordan, 2007

Characteristic	All Participants (n = 3,654), % (SE)	Participants in Household Interview Only (n = 2,889), % (SE)	Participants in Medical Examination (n = 765), % (SE)
**Sex**			
Male	53.1 (0.87)	55.8 (0.94)	42.9 (1.85)
Female	46.9 (0.87)	44.2 (0.94)	57.1 (1.85)
**Age, y**			
18-34	34.5 (0.85)	35.1 (1.00)	31.8 (1.71)
35-49	35.3 (0.87)	35.6 (0.97)	34.1 (1.95)
50-64	19.5 (0.73)	19.0 (0.80)	21.5 (1.80)
≥65	10.8 (0.58)	10.3 (0.64)	12.6 (1.31)
**Education**			
Never attended school	11.4 (0.58)	10.5 (0.64)	14.8 (1.47)
Primary school	32.0 (0.87)	31.8 (0.97)	32.5 (1.96)
Secondary or technical school^a^	42.7 (0.87)	43.4 (0.99)	39.8 (1.83)
University or more	13.9 (0.75)	14.2 (0.88)	12.9 (1.30)
**Current smoker^b^**	29.0 (0.84)	30.3 (0.94)	24.2 (1.76)
**Body mass index,^c^ kg/m^2^**			
25.0-29.9 (overweight)	30.5 (1.86)^d^	NA	30.5 (1.86)
≥30.0 (obese)	36.0 (1.85)^d^	NA	36.0 (1.85)
**Engages in moderate physical activity^e^**	37.8 (0.91)	38.7 (1.05)	34.1 (1.93)
**Typical number of servings of fruits or vegetables per day**			
None	5.3 (0.46)	4.9 (0.50)	6.6 (1.08)
1-4	78.1 (0.84)	78.2 (0.95)	77.5 (1.62)
≥5	16.7 (0.78)	16.9 (0.88)	15.9 (1.50)
**Chronic conditions**			
High blood pressure	17.8 (0.69)	16.4 (0.72)	23.0 (1.84)
High blood cholesterol	7.5 (0.48)	7.1 (0.51)	9.1 (1.29)
Heart disease	8.1 (0.50)	8.2 (0.58)	7.5 (1.00)
Diabetes	9.9 (0.56)	9.4 (0.61)	11.5 (1.41)
Asthma	6.8 (0.44)	6.6 (0.48)	7.7 (1.01)
**Health-related quality of life**			
Fair or poor health status	15.1 (0.64)	13.7 (0.68)	20.4 (1.49)
≥14 unhealthy physical days^f^	6.9 (0.47)	5.9 (0.47)	10.8 (1.18)
≥14 unhealthy mental days^f^	10.3 (0.55)	8.6 (0.54)	16.9 (1.39)
≥14 unhealthy physical/mental days	18.1 (0.73)	15.4 (0.71)	28.4 (1.79)

Abbreviations: CI, confidence interval; OR, odds ratio, SE, standard error.

a Attended or graduated.

b Ever smoked ≥100 cigarettes in a lifetime and currently smoke every day or some days.

c Height and weight measurements to determine body mass index obtained only from participants of the medical examination.

d Values apply only to participants of the medical evaluation.

e Any recreational moderate activity (ie, activity that results in light sweating or small increases in breathing or heart rate).

f During the 30 days prior to the survey.

**Table 2:** Chronic Disease Risk Factors Among Participants in Medical Examination (n = 765), by Sex and Age, Behavioral Risk Factor Surveillance Survey, Jordan, 2007

Risk Factor	Sex, % (SE)		Age, % (SE), y				Total

	Male	Female	18-34	35-49	50-64	≥65	
**Overweight^a^**	35.0 (2.72)	27.1 (2.56)	28.8 (3.56)	31.0 (2.52)	34.8 (3.96)	26.0 (5.08)	30.5 (1.86)
**Obese^a^**	27.4 (2.38)	42.4 (2.51)	18.2 (2.87)	40.5 (2.81)	50.6 (4.63)	43.6 (5.83)	36.0 (1.85)
**High blood pressure^b^**							
Self-reported	20.0 (2.61)	25.3 (2.66)	4.2 (1.33)	12.7 (2.54)	47.1 (4.68)	57.8 (6.02)	23.0 (1.84)
Measured	25.9 (2.35)	16.7 (2.04)	4.1 (1.34)	14.1 (2.33)	42.4 (4.20)	43.2 (5.53)	20.7 (1.45)
Measured + Rx	31.5 (2.79)	28.5 (2.54)	4.5 (1.38)	19.7 (2.65)	61.8 (4.44)	66.3 (5.62)	29.8 (1.79)
**High blood cholesterol^c^**							
Self-reported	6.8 (1.61)	10.8 (1.70)	<1 (0.25)	5.0 (1.42)	17.7 (3.76)	27.4 (5.79)	9.1 (1.29)
Measured	8.2 (1.67)	11.3 (1.62)	4.9 (1.44)	8.0 (1.57)	19.0 (3.36)	12.5 (3.64)	10.0 (1.21)
Measured + Rx	10.9 (1.88)	16.2 (1.98)	5.2 (1.44)	9.1 (1.67)	29.7 (3.84)	22.3 (5.28)	13.9 (1.39)
**Diabetes^d^**							
Self-reported	10.0 (1.80)	12.6 (2.08)	1.1 (0.66)	6.4 (1.94)	28.2 (4.73)	22.3 (3.89)	11.5 (1.41)
Measured	15.0 (2.32)	16.8 (2.20)	5.3 (1.96)	10.4 (2.28)	32.4 (4.18)	30.3 (5.06)	16.0 (1.47)
Measured + Rx	17.9 (2.28)	20.7 (2.26)	6.4 (2.06)	12.5 (2.35)	39.7 (4.57)	37.1 (4.58)	19.5 (1.48)

SE, standard error; Rx, prescribed medication.

a Self-reported height and weight measurements were not obtained in 2007; only physical measures of height and weight were obtained. Overweight defined as a body mass index of 25.0-29.9 kg/m^2^ and obese defined as a body mass index ≥30.0 kg/m^2^.

b Defined as 140/90 mm Hg (systolic/diastolic).

c Defined as ≥240 mg/dL.

d Defined as ≥126 mg/dL.

**Table 3.** Relationship Between Body Mass Index and Selected Health Conditions Among Participants in Medical Examination, Behavioral Risk Factor Surveillance Survey, Jordan, 2007^a^

Health Condition	Body Mass Index (BMI), kg/m^2^		
	<25.0 (Normal, n = 249)	25.0-29.9 (Overweight, n = 235), OR (95% CI)	≥30.0 (Obese, n = 281), OR (95% CI)
**High blood pressure^b^**			
Age-adjusted OR	1 [Reference]	1.87 (1.10-3.19)	2.45 (1.32-4.55)
Fully adjusted OR	1 [Reference]	1.92 (1.11-3.33)	2.85 (1.49-5.44)
**High blood cholesterol^c^**			
Age-adjusted OR	1 [Reference]	1.82 (0.88-3.79)	1.73 (0.89-3.35)
Fully adjusted OR	1 [Reference]	2.23 (1.08-4.62)	2.15 (1.06-4.35)
**Impaired fasting glucose^d^**			
Age-adjusted OR	1 [Reference]	1.21 (0.84-1.76)	1.60 (1.01-2.53)
Fully adjusted OR	1 [Reference]	1.12 (0.78-1.62)	1.43 (0.88-2.33)
**Diabetes^e^**			
Age-adjusted OR	1 [Reference]	1.60 (0.91-2.84)	1.24 (0.65-2.34)
Fully adjusted OR	1 [Reference]	1.52 (0.85-2.71)	1.08 (0.54-2.15)
**Asthma^f^**			
Age-adjusted OR	1 [Reference]	1.16 (0.48-2.77)	1.62 (0.75-3.48)
Fully adjusted OR	1 [Reference]	1.29 (0.55-3.01)	1.71 (0.79-3.70)
**Fair or poor health^f^**			
Age-adjusted OR	1 [Reference]	0.78 (0.43-1.42)	1.32 (0.77-2.29)
Fully adjusted OR	1 [Reference]	0.98 (0.51-1.89)	1.72 (0.99-3.00)

Abbreviations: OR, odds ratio; CI, confidence interval.

a Fully adjusted data are adjusted for sex, age, education, smoking, fruit and vegetable consumption, and physical activity.

b Measured; defined as 140/90 mm Hg (systolic/diastolic).

c Ranged from 0 to 3, with higher scores indicating greater social support.

d Measured; defined as 100-125 mg/dL.

e Measured; defined as ≥126 mg/dL.

f Self-reported.

## Appendix. Jordanian Population by 5-Year Age Group and Sex (Thousands)

Medium Variant 2005^a^

**Table:**

Age, y	Sex		

	**Both**	**Male**	**Female**
0-4	684	350	334
5-9	671	344	327
10-14	614	315	299
15-19	606	312	294
20-24	559	290	269
25-29	481	252	229
30-34	426	226	200
35-39	340	182	158
40-44	255	133	121
45-49	174	88	86
50-54	124	63	61
55-59	111	55	56
60-64	102	51	51
≥65	195	99	92

a Numbers may not sum due to rounding.

Source: Population Division of the Department of Economic and Social Affairs of the United Nations Secretariat, World

Population Prospects: The 2010 Revision,  http://esa.un.org/unpd/wpp/index.htm. Accessed June 1, 2011.
